# Early child health in an informal settlement in the Peruvian Amazon

**DOI:** 10.1186/s12914-016-0099-6

**Published:** 2016-10-12

**Authors:** Gwenyth O. Lee, Maribel Paredes Olortegui, Gabriela Salmón-Mulanovich, Pablo Peñataro Yori, Margaret Kosek

**Affiliations:** 1 Department of Epidemiology, University of Michigan, Ann Arbor, USA; 2 Department of International Health, Johns Hopkins School of Public Health, Baltimore, MD USA; 3 Biomedical Research, Asociación Benéfica PRISMA, Ramirez Hurtado 622, Iquitos, Peru; 4 Independent Researcher, Asociación Peruana para la Conservación de la Naturaleza, Lima, Peru; 5 Department of International Health, Bloomberg School of Public Health, Johns Hopkins University, Room E5608, 615 N. Wolfe Street, Baltimore, MD 21205 USA

**Keywords:** Peru, Informal settlement, Child health, Height-for-age, Natural experiment

## Abstract

**Background:**

Informal settlements are common throughout the developing world. In Peru, land occupations, commonly “invasions” in Spanish, are a means by which the extremely poor attempt to obtain access to land. Here, we examine difference in child health between two communities in the Peruvian Amazon, one well-established and one newly formed by ‘invasion’, as captured incidentally by a prospective epidemiological cohort study.

**Methods:**

Between 2002 and 2006 a study designed to describe the epidemiology of pediatric enteric infections and child growth in a community-based setting enrolled 442 children in *Santa Clara de Nanay*, a community adjacent to the city of *Iquitos*, in Loreto, Peru. In early 2003, a land occupation, commonly called an “invasion” in Spanish, was organized by members of the Santa Clara community, and approximately 20 % of participating study families began occupying privately owned agricultural land adjacent to *Santa Clara*, thus forming the new community of *La Union*.

**Results:**

Parents in families that chose to invade reported less education than parents in families that chose not to. Children in the new community experienced a higher incidence of diarrheal disease and non-specific fevers, although fewer helminth infections, than children who remained in the established community. At the time of the invasion, there were no differences in anthropometric status between the two groups; however children in the new community experienced greater progressive growth faltering over the course of the longitudinal study.

**Conclusions:**

Growth faltering in early childhood represents an enduring loss of human potential. Therefore, our data suggests the human cost of land invasion may be disproportionately borne by the youngest individuals. Innovative policy strategies may be needed to protect this vulnerable group.

## Background

It is estimated that approximately a third of the world’s population [1] and a quarter of Latin Americans [2, 3] now live in informal settlements. These settlements generally develop without formal urban planning, are characterized by dwellings that lack formal legal title, and often lack access to essential public infrastructure [3]. It has long been recognized that a lack of water and sanitation services, crowding, and other conditions of informal settlements are associated with poorer health for both adults and children [4–6]. Because families with legal ownership of their properties become more willing to invest in improvements to their home and community that would lead to improved living conditions [7], and are also more able to engage in economic activities such as more easily renting or mortgaging their properties, which by improving the economic status of the family indirectly leads to improvements in health [8], obtaining legal title to these informal properties has been argued to be an important means of improving child health [9], as well as a source of economic benefit for families [10, 11].

“Invasions” are an important mechanism by which informal settlements in Latin America are formed [12, 13]. Dufour and colleagues describe land invasions as:

“…typically organized at the local level by activists who semi-legally or illegally occupy agricultural or other unoccupied land and sell the lots. They then organize the simultaneous overnight arrival of new land-owners to take possession of their lots by erecting a shelter on the site. These invasions could include hundreds of families…” [12].

Following an invasion, the new landowners then begin the often decades-long process of attempting to obtain legal titles and registration of their lot, which allows them to gain access to public services [3, 11–13]. As regularization proceeds, the health impacts associated with informality may begin to diminish; arguably, the effects of informal settlement on child health will be greatest when the settlement is the newest and least developed.

While poor living conditions in informal settlements are associated with a number of negative impacts on health, including increased burdens of non-communicable disease [6] injuries [4], and infectious diseases such as HIV and tuberculosis [14], the health and development of infants and children in informal settlements has been identified as a key concern [4]. Growth faltering in early childhood is considered to represent an enduring loss of human potential because it is associated not only with higher rates of acute morbidity and mortality [15–17], but also with, in the longer term, poorer cognitive development [18, 19], decreased adult work capacity and earnings [20], and, for girls, a greater risk of poorer maternal health outcomes [15]. While high rates of pediatric diarrheal disease, under-nutrition, and mortality found in recently formed informal settlements have been recognized for a quarter century [5], in general, it is not possible to describe the living conditions of these children prior to their re-settlement, or to estimate the global impact of the living conditions on child health would be relatively to that of their place of origin.

The town of *Santa Clara de Nanay* lies on the *Nanay* River, approximately 15 km from the departmental capital of *Iquitos*, in the department of Loreto, Peru [21]. On February 17th, 2003, a land invasion was organized between members of the *Santa Clara* (SC) community and by members of the nearby community of *Santo Tomas.* A number of families invaded privately owned agricultural land that lay between these two communities, thus forming the informal settlement community of *La Union* (LU). This included a number of families who were at the time participating in a community-based cohort study, begun the year prior, which was designed to describe the epidemiology of pediatric enteric infections and child growth [22]. Here, we describe differences in child morbidity and nutritional status between members of this cohort who lived in SC throughout the study period*,* versus those who initially lived in SC but moved with their families to the new community in 2003.

## Methods

This is a secondary data analysis of an age-stratified, prospective, community-based study of 442 children 0–72 months of age in a semi-rural community in the Peruvian Amazon, between 2002 and 2006. The cohort and study design have been described previously [22–25]. The primary objectives of the original study were unrelated to the results presented here, but related to estimating the incidence of shigellosis in the community, and comparing the association between common etiologies of childhood diarrhea and child growth [22, 25].

Cohort data collection began October 1st, 2002. Only one sibling was eligible to participate in the cohort study at a time and enrollment was ongoing through the study period. As a result, children were followed for variable lengths of time, with some enrolled as early as 2002 and some enrolled as late as 2006. Socio-economic information on the study families was collected through two censuses occurring in February 2002 and May 2005. All study families provided written, informed consent to participate in the study and the study protocol was approved for use by the Institutional Review Boards of the Asociación Benéfica PRISMA in Peru, the U.S. Naval Medical Research Unit No. 6 (NAMRU-6), and Johns Hopkins Bloomberg School of Public Health in Maryland.

Participating families were visited three-times weekly to collect data about illnesses for each participating child; and monthly to collect anthropometry (length or height and weight). Each household was assigned a geographic code indicating its location within the community, and the location in which the child was living within the community was recorded monthly. During episodes of diarrhea, stool samples were collected during or up to two days after the case definition of diarrhea (> = 3 semi-liquid or liquid stools in a 24 h period) was met. The presence of blood in the stool (dysentery) was confirmed by a laboratory technician, and common bacterial enteropathogens including *Shigella*, *Campylobacter*, and Enterotoxigenic *Escherichia coli* (ETEC), and helminthic infections including *Ascaris lumbricoides* and *Trichuris trichiura*, were screened using standard approaches [22, 24]. Episodes of *P. vivax* and *P. falciparum* malaria were subsequently inferred based on the child’s reported use of anti-malarial medication, which was uniquely distributed through the public health care system following thick-smear positivity [23]. Birth weight and birth length was taken from health post records.

Household- and individual level information was complemented with community-level statistics. A community census completed in 2007 (in LU, but not in SC) as part of a study of malaria [26] and a second census conducted in 2010 as part of the study “The Etiology, Risk Factors and Interactions of Enteric Infections and Malnutrition and the Consequences for Child Health and Development” (MAL-ED) were used to provide community-level statistics beyond the timeframe of the original cohort. These censuses were approved as part of their respective study protocols by the Institutional Review Boards of the Asociación Benéfica PRISMA in Peru, and Johns Hopkins Bloomberg School of Public Health in Maryland.

### Statistical methods

The location of the child during each study month was used to construct variables related to the child’s presence in the invasion community. Because a number of study families moved to LU during the initial invasion but stayed only a short time before returning to SC, only families whose location in LU was documented for at least six months of study time were considered to have “participated in the invasion” (LU group) and all other families were considered as part of the “SC group”. LU children who were not under active surveillance at the time of the invasion (i.e. they were enrolled into the cohort later, *N* = 4) were nevertheless assumed to have moved to LU in February, 2003, and their age at the time of the invasion was calculated based on that assumption.

Socio-economic data from the 2002 census (prior to the invasion) and the 2005 census (after the invasion) were compared between SC and LU families. When comparing time-variant characteristics such as access to water, sanitation, and housing type, only children who were already living in 2002, and for whom socio-economic information was available from both 2002 and 2005, were considered.

Differences in morbidity were tested between the two groups using multivariate Poisson regressions with random effects models to account for the correlation between measurements for the same child. The child’s community at the time of the reported illness was included as a predictor of disease incidence. The first set of these models also adjusted for the child’s age at the time of the illness and seasonality, while the second set of models further adjusted for variables related to socio-economic status (per-capita income and mother’s education). Variables related to water and sanitation such as access to a protected water source or an improved latrine were not included, as these proximal determinants of enteric disease were strongly associated with community membership, i.e. membership in the LU community directly affected the participant’s access to protected water sources.

Differences in length-for-age Z-score were considering using linear regression models that included a random intercept to account for correlation between multiple measurements per child. We also constructed regression models where the outcome was the child’s weight and length/height at the cohort close, and the predictors were: total time in the invasion community and presence in the community during the pre-natal, infant and early toddler (<18 months) and early childhood (18–72 months) periods.

Throughout this report, *p*-values of less than or equal to 0.05 were considered statistically significant. No correction for multiple comparisons was used given the small population size and exploratory nature of the analysis. Data analyses were performed using STATA Version 12.1 (StataCorp LP, College Station, TX).

## Results

From 2002 to 2006, the cohort study enrolled 442 children, of whom 433 were included in the analysis presented here. Seven children were excluded due to being present in the study for only a very short (<2 months) period of time. The remaining children contributed a total of 10,985 measures of anthropometry, and 833 complete (after excluding gaps due to travel, etc.) child-months of surveillance data. Of these, 1,477 anthropometric measures (13.1 %), and 114.9 total child-months surveillance (13.8 %) came from children living in the LU invasion community. Eighty children (18.5 %) were documented to be living in the invasion community for at least one month of the study period (from October 2002 to July 2006), 79 (18.24 %) spent at least 3 study months in the community and 71 (16.4 %) spent at least 6 study months in the newly formed community.

Of the 71 children who were documented to be living in LU for at least 6 months during the cohort period, 13 (18.3 %) were unborn at the time of the invasion and were assumed to have spent some portion of their prenatal period, as well as infancy and early childhood in LU. Twenty-three children (32.4 %) were less than 18 months of age at the time of the invasion and therefore spent some part of their infancy and early childhood, but no part of the prenatal period, in the invasion community, and 35 (49.3 %) were between 18 and 72 months of age at the time of the invasion and therefore spent childhood but not infancy in LU.

Families that chose to move to LU were similar in maternal and paternal age, paternal education, and in the percentage of fathers living with the child, to families that did not to participate in the invasion (Tables 1 and 2). However, in 2002, before moving to LU, children in households that later moved had mothers with fewer years of education than those who chose to remain in SC (5.1 versus 6.7 mean years of education, *p*-value = 0.0011), slightly lower per-capita income (51.7 soles/person/month versus 65.8 soles/person/month, *p*-value = 0.0898) (Table 1), were more likely to be living in a household headed by an individual other than the parent of the child (47.2 % non-parent headed versus 32.9 % non-parent headed, *p*-value = 0.0487), and had poorer access to private latrines (61.5 % versus 79.0 %), piped water (47.2 % versus 78.9 %) and fewer homes with improved flooring (5.7 % and 21.0, *p*-value = 0.0085) (Table 2) than families that remained in SC.

**Table 1:** Time-invariant characteristics of SC versus LU families at study enrollment

	Santa Clara	La Union	*p*-value (*t*-test/chi2)
n	362	71	-
% female	49.0 %	46.0 %	0.57
birth weight	3.08 kg	3.09 kg	0.86
birth length	47.0 cm	47.0 cm	0.95
n^a^	353	70	-
Mother’s height	149.3 cm	148.2 cm	0.1462
Mother’s years of education	6.7	5.1	0.0011
Maternal age	26.0	27.8	0.1042
n^a^	204	42	
Paternal height	158.5	159.8	0.6329
Paternal years of education	8.6	7.8	0.2029
Paternal age	31.6	32.2	0.6990

^a^ Each parent counted only once (some families had multiple children in the study); parents not located in either study were not included

**Table 2:** Baseline statistics of the cohort, by location. Only families present in both censuses, with children already living in 2002, are reported here. 329 children in 311 unique households were present in both censuses (results here are presented by household)

	2002 (pre-invasion)			2005 (post-invasion)		
	Santa Clara group	La Union group (still living in SC)	*p*-value (*t*-test/chi2)	Santa Clara group	La Union group (now living in LU)	*p*-value (*t*-test/chi2)
n	258	53	-	258	53	-
% with mother living with child	96.5 %	96.2 %	0.9188	93.8 %	94.4 %	0.8814
% with father living with child	79.5 %	77.4 %	0.7331	82.2 %	95.5 %	0.2587
Identity of head of household = Father	62.0 %	52.8 %	0.2142	68.6 %	73.6 %	0.4750
=Mother	5.4 %	0.0 %	0.0832	3.1 %	3.8 %	0.8011
=Other	32.9 %	47.2 %	0.0487	28.3 %	22.6 %	0.4019
household per-capita income^a^ (soles/person/month)	65.8	51.7	0.0898	61.8	51.4	0.1889
Percent of HHs with Personal Latrine	79.0 %	61.5 %	0.0071	63.6 %	66.0 %	0.7339
Percent of HHs with Piped Water	78.9 %	47.2 %	<0.001	85.3 %	18.9 %	<0.0001
Percent of HHs with Well Water	10.6 %	45.3 %	<0.001	11.6 %	81.1 %	<0.0001
Percent of HHs with metal roof (calamina)	31.9 %	34.0 %	0.7716	36.8 %	15.1 %	0.0021
Percent of HHs with improved floor (wood or cement)	21.0 %	5.7 %	0.0085	26.7 %	5.7 %	0.0009

^a^ geometric means

After moving to LU, access to private latrines was similar between the two groups (66.0 % versus 63.6 %, *p*-value = 0.7339), and differences in the percentage of families headed by a non-parent were also resolved (22.6 % versus 28.3 %). In contrast, the gap in access to piped water and improved housing materials widened (18.9 % versus 85.3 % with piped water, 15.1 % versus 36.8 % with improved roofs, 5.7 % versus 26.7 % with improved floors, in LU and SC, respectively). (Table 2).

The incidence rates of *P. vivax* malaria, non-specific fevers, diarrhea disease, and dysentery were all greater among children living in LU (Table 3); these differences remained significant after adjusting for family socio-economic status (per-capita income and mother’s education). There was no difference in the reported incidence of coughing, and the incidence of the soil-transmitted helminth infections *Ascaris* and *Trichuris* was lower in the invasion community (Table 3).

**Table 3:** The incidence of illnesses between SC and LU communities

	Incidence Rate Ratio^a^ (LU v SC)	*p*-value	Incidence Rate Ratio^b^ (LU v SC)	*p*-value	Incidence Rate SC^c^ (episodes/year)	Incidence Rate LU^c^ (episodes/year)
Vivax malaria	2.26	<0.001	2.14	<0.001	0.17	0.37
Fever (non-specific)	1.11	0.004	1.12	0.003	10.4	11.7
Coughing (non-specific)	1.00	0.980	1.00	0.942	10.3	10.3
Ascaris (detected in any stool sample)	0.79	0.047	0.70	0.003	1.5	0.91
Trichuris (detected in any stool sample)	0.73	0.112	0.59	0.013	0.47	0.01
Dysentery (blood in stool by field workers report)	1.87	<0.001	1.90	<0.001	3.14	5.99
shigellosis	1.42	0.018	1.28	0.107	0.49	0.62
Campylobacteriosis	1.62	0.001	1.60	0.001	0.69	1.13
Giardiasis	1.60	<0.001	1.59	<0.001	1.46	2.48
All cause diarrhea	1.35	<0.001	1.33	<0.001	10.5	14.0

^a^ Based on Poisson models adjusting for child age (as linear splines of age, age > 18 months, and age > 36 months)

^b^ Based on Poisson models adjusting for child age, season, per-capita income, and mother’s education

^c^ Incident rate is based on the estimated incidence at 18 months of age

**Fig. 1:**
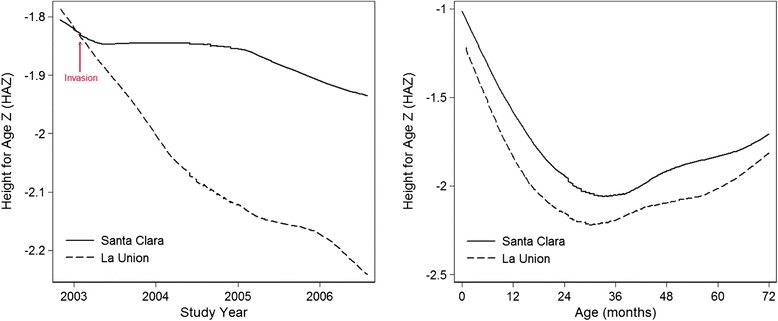
Smoothed height-for-age among children in Santa Clara and La Union, by Age and Study Year. Figures 1a and 2b show the mean smoothed height-for-age of cohort children, by age (Fig. 1a) and study year (Fig. 1b). Overall, children in La Union had a height-for-age 0.14 Z-scores less than those in Santa Clara

**Fig. 2:**
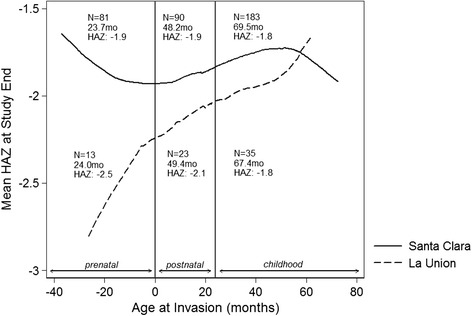
Smoothed height-for-age at the close of the study, among cohort participants based on their age at the time of the invasion. The greatest difference in height-for-age Z occurred between children who were youngest at the close of the cohort (in mid-2006). These children were youngest at the time of the invasion and likely to have spent their pre-natal period, as well as infancy, in the LU community

There were no significant differences in birth weight or birth length between children in the two communities. Children of mothers who likely spent their pregnancies in LU had an average birth length that was 1.6 cm less than children born in SC during the same period (45.8 cm versus 47.3 cm), but this difference was not statistically significant (*p* = 0.247). Overall, during the study period, there was a statistically significant different in height-for-age between the groups, as children in LU had a mean height for age that was 0.14 Z-scores less than children in SC (Fig. 1a). When compared by study time, the difference in height-for-age Z score (HAZ) between the children increased as the study progressed (Fig. 1b). At the close of the cohort study in 2006, the greatest differences in HAZ were between children whose mothers were present in the LU community during the child’s pre-natal development (mean HAZ −2.54 versus −1.87 among children of the same age living in SC, *p*-value = 0.0229), while differences between participants who moved to LU as infants or children and SC children of the same age were non-significant (−2.13 versus −1.91, *p*-value = 0.2627 among the group that moved to LU as infants, and −1.84 versus −1.80, *p*-value = 0.9511 among the group who moved to LU as children) (Fig. 2).

At the 2002 census, prior to the LU invasion, there were 609 households, and 3,472 individuals were accounted for in the community of SC. In 2005, after the invasion, there were 520 households and 2,751 individuals in SC, and 128 households and 587 individuals in LU. Census data from 2007 and 2010 indicate that population of SC subsequently remained stable (554 households and 2,765 individuals accounted for in 2010) while the community of LU continued to grow (181 households in 2007 and 296 households in 2010) (Table 4). Both communities had roughly stabilized by 2012 (an estimated 522 and 283 total households, respectively).

**Table 4:** Difference in housing materials and water/sanitation factors between SC and LU communities over time

	Santa Clara					La Union				
	2002	2005	2007	2010	2012^a^	2002	2005	2007	2010	2012^a^
Number of HHs	609	520	-	554	470	-	128	181	296	255
Number of Individuals	3,472	2,751	-	2,765	2,303	-	587	891	1,263	1,156
Percent of HHs with metal roof (calamina)	173 (28.4 %)	210 (40.4 %)	-	278 (50.2 %)	279 (61.2 %)	-	6 (4.7 %)	16 (8.8 %)	57 (19.3 %)	98 (38.74 %)
Percent of HHs with improved floor (wood or cement)	126 (20.7 %)	174 (33.5 %)	-	198 (35.7 %)	205 (45.0 %)	-	4 (3.1 %)	12 (6.6 %)	3 (11.8 %)	58 (22.9 %)
Percent of HHs with brick/cement exterior walls	104 (17.1 %)	104 (20.0 %)	-	177 (32.0 %)	117 (25.6 %)	-	0 (0.0 %)	2 (1.1 %)	6 (2.0 %)	11 (4.4 %)
Percent of HHs with Personal Latrine	452 (74.2 %)	329 (63.3 %)	-	357 (64.4 %)	312 (68.4 %)	-	85 (66.4 %)	121 (66.9 %)	200 (67.6 %)	164 (64.4 %)
Percent of HHs with Piped Water	447 (73.4 %)	447 (96.0 %)	-	511 (92.2 %)	392 (85.8 %)	-	7 (5.5 %)	2 (1.1 %)	1 (0.3 %)	7 (2.8 %)
Percent of HHs with Well Water	20 (3.3 %)	60 (11.5 %)	-	33 (6.0 %)	57 (12.5 %)	-	121 (94.5 %)	175 (96.7 %)	295 (99.7 %)	7 (2.8 %)

^a^ The 2012 census had a slightly higher refusal rate compared to earlier censuses, and was also conducted during the period when much of the community typically travels, so these numbers may be a relative under-estimate

The prevalence of improved housing materials such as metal roofs and cement floors improved from 2002 to 2012 in both communities, while the prevalence of personal latrines, and access to piped water, remained relatively constant (Table 4).

## Discussion

We found that being physically present in a newly established informal settlement was associated with poorer child health. Children in LU had poorer nutritional status (HAZ) than children who remained in the SC community, and were also at increased risk of *P. vivax* malaria, non-specific fevers and diarrheal disease, as well as shigellosis and dysentery specifically. Because malaria and dysentery are associated with increased rates of hospitalization and mortality, particularly among undernourished children [16, 27], these diseases are a particular cause for concern.

It is likely that the elevated incidence of diarrheal disease in the invasion cohort was the result of decreased access to safe drinking water, rather than differences in access to sanitation, in the first years following the invasion. In SC, most households had access to piped, chlorinated drinking water, whereas in LU unprotected wells were the main source of drinking water. Although families that later chose to invade had poorer access to improved water sources even before leaving SC, this access was reduced even further once they moved to LU. In contrast, neither community had a sewage system and both communities reported fairly similar access to latrines, so this is less likely to have been a source of difference in exposure between the sites. Cement floors are increasingly recognized as a potentially important source of protection against enteropathogens [28], and this may also have contributed to differences in the incidence of disease between the two communities. The percentage of households reporting private latrines in SC decreased from 2002 to 2005. Simultaneously, reported latrine sharing increased. However, the underlying cause of this shift is unknown.

The relatively low prevalence of soil-transmitted helminthic infections in LU is likely related to environmental differences between the communities. Helminthes are transmitted when eggs present in human feces contaminate the soil, and tend to be present in inhabited areas where sanitation is poor. As a new site of human settlement, the soil in LU was likely initially uncontaminated by these species.

LU families were less educated than SC families, and, although not statistically significant, their incomes were marginally lower. Because maternal education has consistently been associated with higher burdens of disease and poorer nutritional status within communities, it is likely that these children would also have experienced relatively higher rates of disease and undernutrition had they remained in the original community. However, several factors make it unlikely that this explains all of the difference observed. First, risk factors for enteric diseases are well-studied, and access to safe drinking water is an important protective factor that LU lacked. Secondly, differences in the incidence of disease between the communities remained significant after controlling for family socio-economic factors by the inclusion of per-capita income and maternal education in our models. The association between family socio-economic status and child morbidity is mediated through multiple mechanisms, some of which (e.g. access to improved water) are more likely to have been affected by the invasion than others (e.g. food security and dietary intake, although these factors were not measured in this study). Therefore it seems likely that, had LU families chose to remain in SC, the rates of morbidity that their children experienced would have been between those of the families that chose not to invade, and what was actually observed.

These families, who chose to move over a very short distance, may be compared in some respects to individuals who chose to migrate, for example, rural-to-urban migrants or individuals who migrate internationally. Many studies have found that migrants tend to be in better health that those who chose not to migrate, termed “healthy migrant” [29, 30]. This is hypothesized to be due to self-selection and to explain, for instance, the lower rates of infant mortality among the migrant mothers [31]. This same self-selection might also be present among invaders. However, our data suggests that the disadvantages to child health associated with the informal settlement environment outweigh any potential “healthy invader” bias that may exist among pregnant women and their offspring. The pre-natal and infant periods are critical for human development, and disadvantages suffered during these periods are likely to have life-long consequences. Later wealth does not negate the disadvantages of poorer socioeconomic status in early life [32], and even stunted toddlers who experience catch-up in growth at later ages [33, 34] may not experience ‘catch-up’ in cognitive development [35]. Therefore it is unlikely that any economic benefits that may accrue to the family subsequently, as a result of their involvement in the invasion, will fully counter-balance these early developmental disadvantages.

There are a number of limitations to our study. This study was not designed with the intention of examining the health of children during an invasion as a primary or secondary outcome. Rather, the invasion led to a natural experiment that the cohort study happened to capture. As such, the results we present here are purely descriptive. Nevertheless, detailed longitudinal surveillance data documenting the incidence, prevalence, and etiology of childhood illnesses during and immediately after the formation of an informal settlement is unusual and therefore worthy of consideration. Community level statistics in the decade following the study suggest that differences in the burden of disease may have decreased but not fully resolved in the decade following the invasion, since by 2012 LU still had not achieved significant access to improved water sources. The diagnosis of malaria was based on the child’s reported use of anti-malarial medication, which although uniquely distributed through the public health care system following thick-smear positivity may have underestimated the true incidence of this disease, particularly in LU which was slightly more distant to the health center. An additional limitation is the relatively small number of children in the LU group, and, in particular, the small number of children who were very young at the time of the invasion. Overall, there were statistically significant differences in the height-for-age of children between the two communities, and stratified analyses by age seem to suggest that most of this difference was concentrated among those who lived in the invasion community in infancy, while early childhood may have been a less critical period. However, we lack the sample size to investigate these differences more fully within out sample.

Unfortunately, no information was gathered as to the motivations or intentions of the invaders, or how the health of their children may have affected their decisions. Anecdotally, many of the invaders were young parents eager to form independent households, which our data seems to support. Additionally, parents also appear to weigh the health of their children carefully in making their decisions to invade: because of the risk of expulsion from the informal settlement by police, they are concerned about the risk of violence to their children; and parents may send their children to live with extended family or neighbors rather than bring them into the new community. However, children are also regarded as an asset to an invasion insofar as police are considered less willing to forcibly evict a community with many families.

Arguments about public health are frequently wielded in policy debate related to informal settlements in Latin America as well as globally. While our findings support a negative association between newly-formed informal settlements and child health, Peru has strong policies promoting the formalization of land ownership [11], and there is evidence that, in the longer term, land ownership is associated with improved child nutrition status, especially in rural areas [9, 36]. Slum upgrading strategies, including improvements to water and sanitation, energy, and transportation infrastructure, and housing improvements such as improved flooring are also associated with less childhood illness [37]. However, a recent Cochrane review also highlighted the need for qualitative and quantitative effectiveness data to better determine which strategies should be prioritized in order to have the greatest health impact [37].

However, because new communities are constantly formed, and then gradually achieve formality and greater access to services, families are, in effect, trading years living under basic conditions for the opportunity of later home ownership. In this early phase of the community lifecycle, household level strategies to promote water treatment and storage should be prioritized to protect child health [38]. At a policy level, there is also a need for urban development strategies that create pathways to land ownership for poor families that do not disadvantage the health of their children.

## Conclusion

The Peruvian economist Hernando de Soto has written that “contrary to what one might believe, invaders pay a very high price for the land they occupy. Since they have no money, they pay for it with their own human capital” [8]. The pre-natal and infant periods are critical for human development, and disadvantages suffered during these periods are likely to have life-long consequences. Our study describes a high burden of morbidity and undernutrition among the young children of families occupying a new area. Despite the study limitations, this study agrees with previous findings regarding the impact of living conditions on child health, but also adds a quantitative estimate of how much all previous recognized insults work together in an informal settlement to exert pressure on child development. We expect these findings can help measure the effect of specific policies directed to prevent informal settlements and their improvement considering the undeniable costs in human capital.

## Abbreviations

ETEC: Enterotoxigenic *Escherichia coli*
HAZ: Height-for-age Z score
LU: La Union
SC: Santa Clara

## References

[1] UN-Habitat (2012). Making slums history- a global challenge for 2020.

[2] Mac Donald J (2004). Pobreza Y Precariedad Del Hábitat En Ciudades de América Latina Y El Caribe.

[3] Fernandes E. Regularization of Informal Settlements in Latin America. Cambridge: Lincoln Institute of Land Policy; 2011.

[4] Sverdlik A (2011). Ill-health and poverty: a literature review on health in informal settlements. Environ Urban.

[5] de Romaña GL, Brown KH, Black RE (1987). Health and growth of infants and young children in Huáscar, Perú. Ecol Food Nutr.

[6] Heitzinger K, Montano SM, Hawes SE, Alarcón JO, Zunt JR (2014). A community-based cluster randomized survey of noncommunicable disease and risk factors in a peri-urban shantytown in Lima, Peru. BMC Int Health Hum Rights.

[7] Besley T (1995). Property rights and investment incentives: theory and evidence from Ghana. J Polit Econ.

[8] de Soto H (1987). El Otro Sendero: La Revolucion Informal.

[9] Galiani S, Schargrodsky E (2004). Effects of land titling on child health. Econ Hum Biol.

[10] Galiani S, Schargrodsky E (2010). Property rights for the poor: effects of land titling. J Public Econ.

[11] Lastarria-Cornhiel S (1999). Formalizing informality : the praedial registration system in Peru.

[12] Dufour DL, Piperata B (2004). Rural-to-urban migration in Latin America: an update and thoughts on the model. Am J Hum Biol.

[13] Dosh P (2010). Demanding the land: urban popular movements in Peru and Ecuador, 1990–2005.

[14] David AM, Mercado SP, Becker D, Edmundo K, Mugisha F (2007). The prevention and control of HIV/AIDS, TB and vector-borne diseases in informal settlements: challenges, opportunities and insights. J Urban Heal.

[15] Black RE, Allen LH, Bhutta ZA, Caulfield LE, de Onis M, Ezzati M, Mathers C, Rivera J (2008). Maternal and child undernutrition: global and regional exposures and health consequences. Lancet.

[16] Caulfield LE, Richard SA, Black RE (2004). Undernutrition as an underlying cause of malaria morbidity and mortality in children less than five years old. Am J Trop Med Hyg.

[17] Caulfield LE, de Onis M, Blössner M, Black RE, Blossner M (2004). Undernutrition as an underlying cause of child deaths associated with diarrhea, pneumonia, malaria, and measles. Am J Clin Nutr.

[18] Walker SP, Grantham-Mcgregor SM, Powell CA, Chang SM (2000). Effects of growth restriction in early childhood on growth, IQ, and cognition at age 11 to 12 years and the benefits of nutritional supplementation and psychosocial stimulation. J Pediatr.

[19] Grantham-Mcgregor S, Cheung YB, Cueto S, Glewwe P, Richter L, Strupp B (2007). Child development in developing countries 1: developmental potential in the first 5 years for children in developing countries. Lancet.

[20] Haas JD, Murdoch S, Rivera J, Martorell R (1996). Early nutrition and later physical work capacity. Nutr Rev.

[21] Yori P, Lee G, Olórtegui MP, Trigoso DR, Flores JT, Vasquez AO, Rengifo JC, Burga R, Pinedo SR, Asayag CR, Caulfield L, Kosek M (2014). Santa Clara de Nanay: the MAL-ED cohort in Peru. Clin Infect Dis.

[22] Kosek M, Yori P, Pan WK, Olortegui MP, Gilman RH, Perez J, Chavez CB, Sanchez GM, Burga R, Hall E (2008). Epidemiology of highly endemic multiply antibiotic-resistant shigellosis in children in the Peruvian Amazon. Pediatrics.

[23] Lee G, Peñataro Yori P, Paredes Olortegui M, Pan W, Caulfield L, Gilman RH, Sanders JW, Silva Delgado H, Kosek M (2012). Comparative effects of vivax malaria, fever and diarrhoea on child growth. Int J Epidemiol.

[24] Lee G, Pan W, Yori PPP, Olortegui MP, Tilley D, Gregory M, Oberhelman R, Burga R, Chavez CB, Kosek M (2013). Symptomatic and asymptomatic campylobacter infections associated with reduced growth in Peruvian children. PLoS Negl Trop Dis.

[25] Lee G, Paredes Olortegui M, Yori P, Black RE, Caulfield LE, Banda Chavez C, Hall E, Pan WK, Meza R, Kosek M (2014). Effects of shigella, campylobacter, and ETEC-associated diarrhea on childhood growth. Ped Infect Dis J.

[26] Chuquiyauri R, Paredes M, Peñataro P, Torres S, Marin S, Tenorio A, Brouwer KC, Abeles S, Llanos-Cuentas A, Gilman RH, Kosek M, Vinetz JM (2012). Socio-demographics and the development of malaria elimination strategies in the low transmission setting. Acta Trop.

[27] Bhutta ZA, Ahmed T, Black RE, Cousens S, Dewey K, Giugliani E, Haider BA, Kirkwood B, Morris SS, Sachdev HPS, Shekar M (2008). What works? Interventions for maternal and child undernutrition and survival. Lancet.

[28] Cattaneo MD, Galiani S, Gertler PJ, Martinez S, Titiunik R (2009). Housing, health, and happiness. Am Econ J.

[29] Razum O, Zeeb H, Rohrmann S (2000). The “healthy migrant effect”—not merely a fallacy of inaccurate denominator figures. Int J Epidemiol.

[30] Rubalcava LN, Teruel GM, Thomas D, Goldman N (2008). The healthy migrant effect: new findings from the Mexican Family Life Survey. Am J Public Health.

[31] Wingate MS, Alexander GR (2006). The healthy migrant theory: variations in pregnancy outcomes among US-born migrants. Soc Sci Med.

[32] Krishna A, Oh J, Lee J, Lee H-Y, Perkins JM, Heo J, Ro YS, Subramanian SV (2015). Short-term and long-term associations between household wealth and physical growth: a cross-comparative analysis of children from four low- and middle-income countries. Glob Health Action.

[33] Outes I, Porter C (2013). Catching up from early nutritional deficits? Evidence from rural Ethiopia. Econ Hum Biol.

[34] Lourenço BH (2012). Determinants of linear growth from infancy to school-aged years : a population-based follow-up study in urban Amazonian children Keywords. BMC Public Health.

[35] Sokolovic N, Selvam S, Srinivasan K, Thankachan P, Kurpad aV, Thomas T (2014). Catch-up growth does not associate with cognitive development in Indian school-age children. Eur J Clin Nutr.

[36] Victora CG, Vaughan JP, Kirkwood B, Martines JC, Barcelos LB (1986). Child malnutrition and land ownership in Southern Brazil. Ecol Food Nutr.

[37] Turley R, Saith R, Bhan N, Rehfuess E, Carter B. Slum upgrading strategies involving physical environment and infrastructure interventions and their effects on health and socio-economic outcomes. CochraneDatabase of Systematic. 2013;(1):CD010067. doi:10.1002/14651858.CD010067.pub2/and infrastructure interventions and their effects on health and socio-economic outcomes. Cochrane. 2013. 10.1002/14651858.CD010067.pub2 PMC12423365 23440845

[38] WHO (World Health Organization). Combating waterborne disease at the household level. 2007.

